# Standardized Informed Consent Form for Clinicians Administering Platelet-Rich Plasma

**DOI:** 10.7759/cureus.57565

**Published:** 2024-04-03

**Authors:** Satvik N Pai, Naveen Jeyaraman, Ravichandran Venkatasalam, Ravi VR, Swaminathan Ramasubramanian, Sangeetha Balaji, Arulkumar Nallakumarasamy, Shilpa Sharma, Bishnu P Patro, Madhan Jeyaraman

**Affiliations:** 1 Orthopaedic Surgery, Hospital for Orthopedics, Sports Medicine, Arthritis & Trauma (HOSMAT) Hospital, Bangalore, IND; 2 Orthopaedic Surgery, Sri Ramachandra Institute of Higher Education and Research, Chennai, IND; 3 Orthopaedics, ACS Medical College and Hospital, Dr MGR Educational and Research Institute, Chennai, IND; 4 Regenerative Medicine, Orange Healthcare, Chennai, IND; 5 Regenerative Medicine, Mothercell Regenerative Centre, Tiruchirappalli, IND; 6 Orthopaedics, Government Medical College, Omandurar Government Estate, Chennai, IND; 7 Orthopaedics, Jawaharlal Institute of Postgraduate Medical Education and Research (JIPMER) – Karaikal, Karaikal, IND; 8 Pediatric Surgery, All India Institute of Medical Sciences, New Delhi, IND; 9 Orthopaedics, All India Institute of Medical Sciences, Bhubaneswar, IND; 10 Clinical Research, Viriginia Tech India, Dr MGR Educational and Research Institute, Chennai, IND

**Keywords:** consent, medico-legal, prp, platelet-rich plasma, informed consent

## Abstract

Introduction

When it comes to medico-legal malpractice suits, lawyers and insurers tend to focus on informed consent documentation. Unfortunately, there is no standard protocol for obtaining informed consent for the use of platelet-rich plasma (PRP) injections, which might cause problems. This study aimed to mitigate this concern through the development of a standardized informed consent document for PRP injections, grounded in evidence-based practices.

Materials and methods

An examination of databases was conducted to explore the medico-legal ramifications associated with PRP injections, as well as the broader topic of informed consent, with a particular focus on the context of PRP injections. Moreover, interviews were carried out with healthcare providers and individuals who had received PRP injections within the preceding year, utilizing a semi-structured methodology.

Results

We developed an evidence-based informed consent document tailored for PRP injections. To guarantee its legal validity, the document underwent review by a legal specialist. Subsequently, our institutions implemented the finalized form for PRP injection procedures over one year.

Conclusion

A legally valid and evidence-based informed consent form for PRP injections would ensure patient's rights, and encourage open communication and transparency between them and the doctor. Moreover, if a lawsuit were to arise, it would serve as a critical document in the doctor's defense and withstand scrutiny from lawyers and the judiciary.

## Introduction

Informed consent is a crucial aspect of medical ethics and patient autonomy, as it enables patients to participate in their own healthcare decisions. For several reasons, obtaining informed consent from a patient before a medical procedure is crucial. It ensures that the patient is fully aware of the risks, benefits, and alternatives to the procedure, and can make an informed decision about their healthcare. Informed consent helps to establish trust and rapport between the patient and the healthcare provider, leading to better outcomes and higher patient satisfaction. Moreover, it provides legal protection for the healthcare provider in case of any adverse events or complications. In recent decades, the importance of informed consent has markedly increased, emerging as a pivotal aspect in numerous legal disputes. It frequently serves as the primary focus for legal representatives and insurers in cases of medical-legal malpractice.

Platelet-rich plasma (PRP) injections have become popular in recent years. PRP injections involve the use of the patient own blood component, which is drawn and processed to concentrate platelets and growth factors [1]. PRP demonstrates a broad spectrum of applications within dermatology, notably in hair restoration, skin rejuvenation, addressing acne scars, and dermal augmentation. Evidenced benefits extend to conditions such as alopecia, pigmentary disorders, lichen sclerosis, leprosy-induced peripheral neuropathy, plaque psoriasis, and nail disorders [2]. Moreover, the synergistic effects achieved by integrating PRP with laser therapies, microneedling, dermal fillers, and autologous fat grafting contribute to enhanced aesthetic outcomes [3]. PRP injections are used in other medical fields as well. It has been used in orthopedics to treat conditions such as osteoarthritis, tendonitis, ligament sprains, and muscle strains and to promote bone healing after fractures [4-7]. PRP also has applications in urology for the treatment of erectile dysfunction and Peyronie’s disease [8].

The absence of a consistent and established method for acquiring informed consent for the administration of PRP is a concern [9]. Consequently, the omission of critical information in informed consent documents for the procedure poses a risk to their legal integrity in court. This highlights the necessity of creating a predefined, evidence-backed informed consent form specifically designed for the administration of PRP injections [10].

## Materials and methods

**Figure 1:**
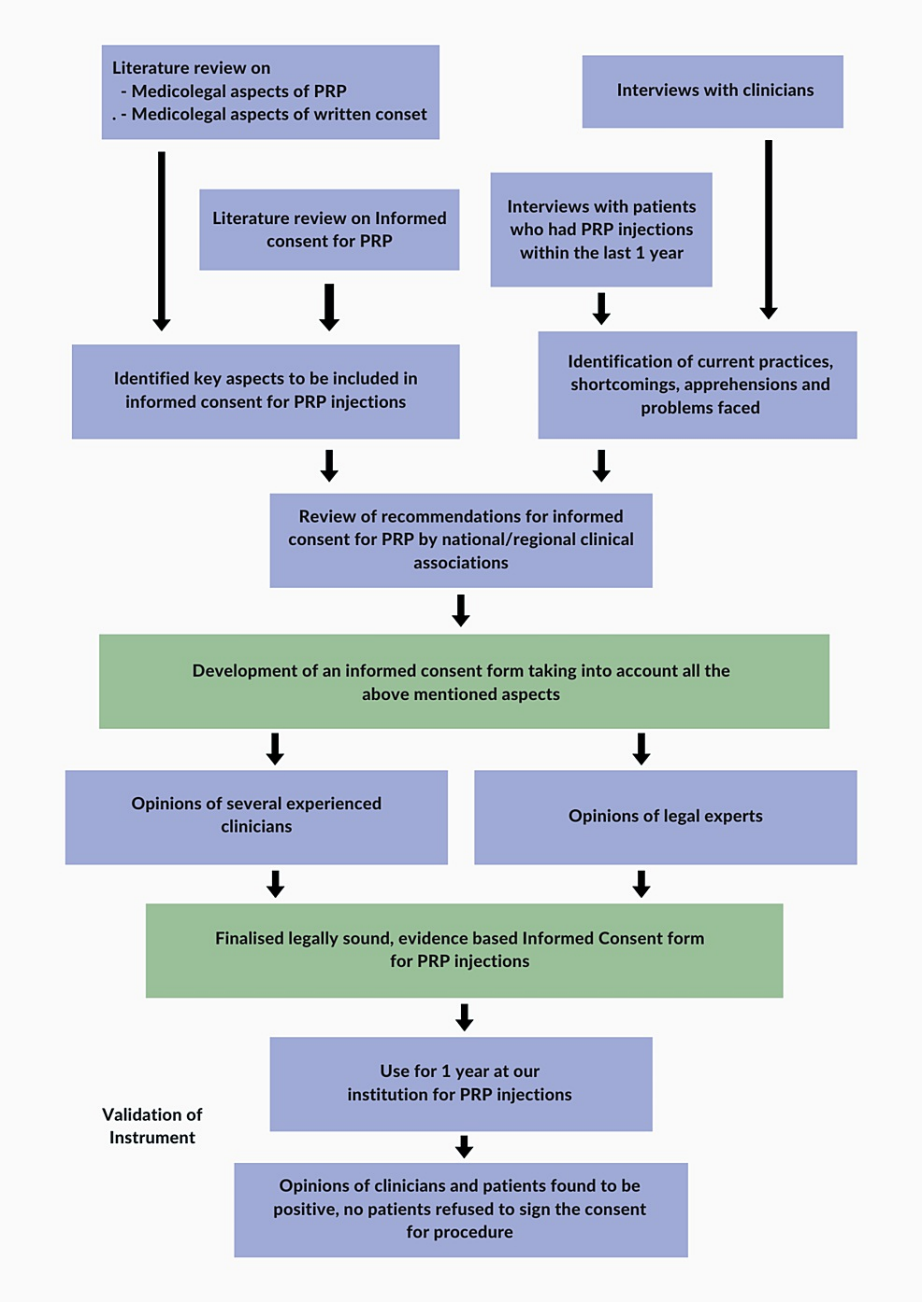
Flowchart illustrating the process entailed in the development of the informed consent document PRP: platelet-rich plasma

Upon obtaining ethical approval from the Faculty of Medicine of the Sri Lalithambigai Medical College and Hospital, Chennai, India (approval Dr MGR-ERI/SLMCH/2022/012), an extensive review of the literature on informed consent for PRP injections was conducted, including medico-legal aspects and complications. Using medical subheading terms and Boolean operators, PubMed and Cochrane Library databases were screened by hand. Additionally, we conducted searches across online legal databases and literature, seeking medico-legal cases related to PRP injection instances in legal courts, consumer dispute resolution forums, and state medical councils. The outcomes of our literature review were meticulously documented and organized. Furthermore, we engaged in semi-structured interviews with both clinicians and patients who had received PRP injections within the preceding year. We asked the clinicians about common practices and difficulties related to informed consent for PRP injections, as well as patient concerns that they encountered. We asked the patients about their experiences with the informed consent process, its usefulness, and any doubts that were not satisfactorily addressed. Drawing upon this data, we formulated an evidence-based informed consent document, which was subsequently reviewed by seasoned clinicians and a legal expert to gather additional feedback. We made minor modifications based on their suggestions and prepared the final consent form (Figure 1). This consent form was used for 172 patients at our institutions without any concerns raised or patients refusing to sign. We did not find the need for any additional modifications. Both clinicians and patients responded positively to the consent form.

Validation of instrument

The study engaged a multidisciplinary panel of experts, comprising internal and external members with specialized knowledge in various medical fields. Internal experts included three individuals from the Departments of Dermatology, Orthopedics, and Dentistry, along with one expert each from the Departments of Pharmacology and Community Medicine, all based in Chennai. The external expert panel was diverse, featuring individuals from prestigious institutions across India. These experts were provided with the study's tools, objectives, blueprints, and a criteria rating scale for comprehensive assessment and feedback. After validating the content, all the tools were returned.

Baseline proforma of the study participants

The majority of the items in the informed consent proforma were agreed upon unanimously. The proforma consisted of 20 items for study participants, but some modifications were made based on the suggestions of the validators. After expert consultation, two items had less than 60% agreement and were subsequently removed. Consequently, the informed consent form for the present study had 18 items.

To evaluate the content validity ratio (CVR), the questionnaire was disseminated to six specialists with expertise relevant to the study's domain. Responses were structured using a three-point Likert scale, categorizing items as essential, helpful but not essential, or unnecessary. Subsequently, the CVR of the questionnaire was assessed, with items scoring above 0.95 deemed essential and pertinent according to the Lawsche table. Items receiving lower scores, those deemed ineffective in measuring the intended concept, or those lacking relevance to the topic, were excluded based on feedback from experts and respondents' comments.

To examine the indexes of 'relevance', 'clarity', 'simplicity', and 'ambiguity', experts were asked to provide their opinions and suggestions regarding the items that should be included in the questionnaire. A separate content validity index (CVI) was calculated for each individual item and scale. Therefore, we calculated the scale-content validity index (S), where, $S = CVI/Average$, for the four constructs (relevance, clarity, simplicity, ambiguity) to be 0.94, using the experts' responses and suggestions [11].

## Results

**Figure 2:**
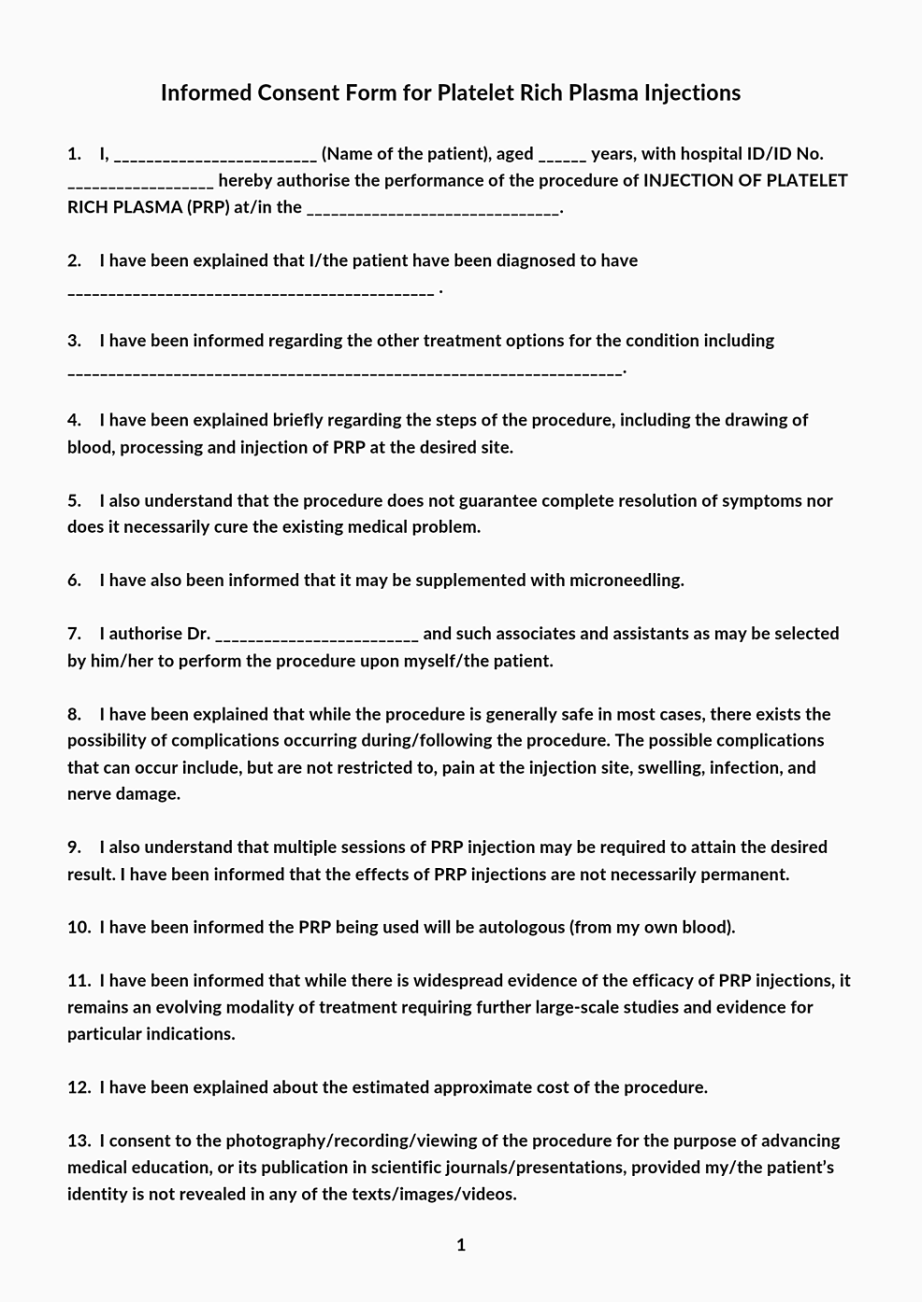
A standardized informed consent form for clinicians administering platelet-rich plasma injections, Page 1

**Figure 3:**
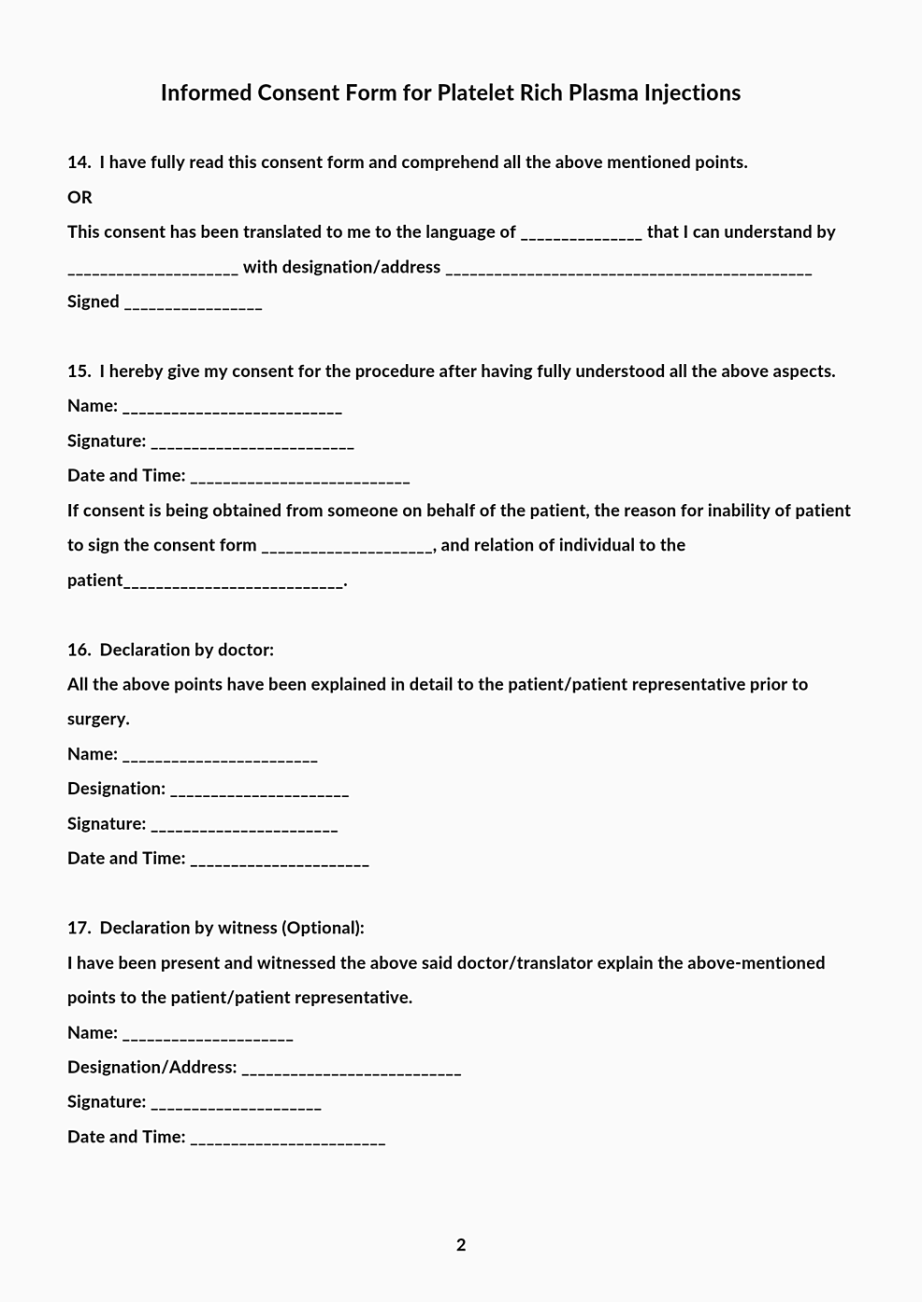
A standardized informed consent form for clinicians administering platelet-rich plasma injections, Page 2

A standardized informed consent form for clinicians administering platelet-rich plasma injections is given in Figure 2 and Figure 3.

## Discussion

According to the literature, informed consent is crucial in defending against malpractice claims, however, studies suggest that the informed consent process among clinicians and the understanding among patients is incomplete and needs improvement [12,13]. This incomplete informed consent can leave clinicians at risk for malpractice claims, which can be used against them in court. The use of generic forms for different procedures is insufficient as they do not provide adequate documentation of complications that could arise from the procedure. Instead, procedure-specific consent forms are recommended [14]. Documenting the patient's diagnosis and thoroughly discussing all alternative treatment options before surgery is crucial, as this aspect is frequently neglected in informed consent documentation [15]. It is also essential to explain the procedure to the patient in a way that is easy to understand, and for the doctor to discuss the goals of the procedure to manage expectations [16]. Moreover, patients should be informed that the procedure may not result in complete resolution of their symptoms.

**Figure 4:**
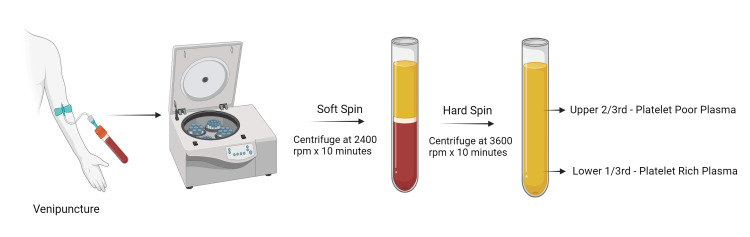
Preparation of Platelet Rich Plasma (PRP) RPM - Rotations per minute

PRP injections are generally regarded as a very safe procedure, with major adverse effects being rare occurrences [17]. The procedure for preparation of PRP is explained briefly in Figure 4. However, it is important to inform patients of the possible common complications and any rarer, serious complications that may arise. Given the frequent absence of consensus among clinicians regarding the inclusion of complications in informed consent documents, it is prudent to adhere to guidelines established by a national or regional association of clinicians. Unfortunately, such recommendations are lacking in many countries, which has resulted in an absence of standard practice and uniformity. To address this issue, our consent form lists all relevant complications based on available literature and common causes of litigation in PRP injection cases [18-24]. Additionally, it is often overlooked that patients should also be counselled regarding the possible need for multiple injections and supplementation with other forms of treatment, as this is important information for the patient to be aware of following the procedure [25]. Another unique aspect of obtaining informed consent for PRP injections is that the use of PRP has only become popular within the last two decades, and may not yet be universally accepted among medical professionals [26-28]. Therefore, we have included a clause in our consent form to acknowledge this fact. It is advisable to seek consent in advance for the documentation of surgical procedures through photography or recording, particularly for educational or publication purposes in scientific journals, as an integral component of research ethics [29].

Facilitating informed consent for non-English-speaking patients through the provided consent form may present legal challenges. To ensure the legitimacy of consent in such cases, it is advisable to record the translated language, pertinent translator information, and their endorsement. Any individual proficient in both English and the patient's language may serve as the translator. Essential elements of the consent process include signatures from the patient and the physician, while having a witness sign the form is suggested though not mandated by law. As the consent form currently exists solely in English, undertaking cross-cultural validations is imperative to offer it in additional languages.

This consent form is based on medical evidence and legal review, but its objective assessment in terms of law is hardly possible. A legal trial analyzing and discussing this consent form can make it more reliable, but even then it will only be valid for that specific case. That being said, it is always better to be prepared and comply with the law, and we believe that this consent form will be useful in that regard.

## Conclusions

The development of a standardized informed consent form for PRP injections marks a pivotal advancement in enhancing patient-provider relationships and legal safeguards across dermatology, orthopedics, and more. By meticulously incorporating medical evidence, expert insights, and legal considerations, this consent form significantly bolsters patient autonomy, fortifies trust, and minimizes legal vulnerabilities. It meticulously addresses PRP treatment's unique characteristics and its integration with other therapies, ensuring patients are thoroughly informed about their options, potential outcomes, and risks. The form's proven effectiveness in clinical settings, evidenced by positive patient and clinician feedback, highlights its contribution to the informed consent process. Future efforts should aim at its cross-cultural validation and adaptation, furthering our commitment to patient-centered care and ethical medical standards.
